# Genomics-Driven Mulberry Breeding for Improving Agronomic Traits and Circular Utilization Value

**DOI:** 10.3390/biology15080628

**Published:** 2026-04-16

**Authors:** Yanmei Wang, Chenfeng Yang, Xiaofeng Li, Ruojin Liu, Shuaishuai Huang, Yonghong Zhou

**Affiliations:** 1 Key Laboratory of Biodiversity and Environment on the Qinghai-Tibetan Plateau, Ministry of Education, Xizang University, Lhasa 850000, China; 18387088190@163.com (Y.W.); 13198663710@163.com (C.Y.); daiilxf@163.com (X.L.); 13244000808@163.com (R.L.); 2 School of Ecology and Environment, Xizang University, Lhasa 850000, China

**Keywords:** mulberry genomics, breeding technologies, stress resistance, multi-omics, circular utilization

## Abstract

This review systematically summarizes the history of and recent progress in mulberry research, with particular emphasis on advances in mulberry genomics and its role in modern breeding and the circular bioeconomy. Studies on whole-genome sequencing, high-density genetic mapping, and comparative genomics are reviewed. In addition, strategies for identifying functional genes associated with stress resistance, quality traits, and metabolic pathways are summarized. Building on these advances, the applications of genome editing, marker-assisted selection, and multi-omics-integrated breeding for improving stress resistance in mulberry are discussed. Current evidence indicates that developments in genomics have significantly shortened the mulberry breeding cycle, improved selection precision, and enhanced breeding efficiency while also providing molecular support for the development of a mulberry-based circular bioeconomy chain. In recent years, substantial progress has been achieved in mulberry genomics research. However, as an allopolyploid plant, the complexity of the mulberry genome continues to pose challenges for in-depth genomic analysis. Current limitations include incomplete reference genomes, insufficient functional annotation, and complex genetic backgrounds that hinder further genomic dissection. To address these challenges, strategies such as multi-omics integration, optimization of emerging genome-editing technologies, and diversified application models are proposed. These approaches aim to promote high-quality precision breeding and strengthen the integration of mulberry improvement with the circular bioeconomy, thereby maximizing the utilization and application value of mulberry resources.

## Simple Summary

Mulberry is an important economic crop widely distributed around the world. It has attracted considerable attention because of its value in food production, medicinal applications, and ecological restoration. This review examines the role of genomic advances in promoting mulberry breeding. The development history and recent research progress in mulberry breeding are systematically summarized, with particular emphasis on the role of rapidly advancing genomic technologies in modern breeding strategies. Applications of traditional breeding, genome editing, marker-assisted selection, and multi-omics-integrated breeding for improving stress resistance in mulberry are highlighted. Advances in genomics have significantly shortened the mulberry breeding cycle, improved selection precision, and enhanced breeding efficiency. In addition, current bottlenecks and challenges in the genomic breeding of mulberry are analyzed in light of recent research progress. Finally, prospective strategies, including multi-omics integration and diversified breeding strategies, are proposed to address existing limitations. This review aims to provide useful insights and references for the innovation and diversification of mulberry germplasm resources.

## 1. Introduction

Mulberry (*Morus* L., Moraceae) is a perennial tree that has been cultivated for more than 7000 years [1]. The genus comprises approximately 30 species and 10 varieties and is widely distributed across tropical, subtropical, and temperate regions of East, Southeast, and Southwest Asia, Oceania, southwestern Africa, southern Sudan, Madagascar, southern North America, Central America, and western South America. China possesses the richest mulberry genetic resources in the world, including 15 species, 4 varieties, and 7000 cultivated varieties [2,3,4]. Studies based on molecular phylogeny and geographic distribution indicate that the mulberry most likely originated in China’s Yarlung Tsangpo River basin [5]. Approximately 5000 years ago, ancient Chinese communities recognized its high protein productivity and capacity to attract wild silkworms. The improvement of mulberry and the domestication of wild silkworms subsequently led to the development of sericulture and silk production, profoundly influencing China’s economic, cultural, and medical development [6]. As society advances, the mulberry tree’s exceptional ecological adaptability has become increasingly evident, and its ecological and economic value is now widely recognized. Beyond its traditional role in sericulture, the mulberry has gained significant attention for its potential applications in ecological restoration, feed development, functional foods, and medicinal resources—underscoring its considerable research value and broad application prospects [7].

As an important economic crop, mulberry has played a significant role throughout human history. In recent years, with increasing global emphasis on sustainable development, mulberry research and applications have become important contributors to the transition toward a circular bioeconomy [8]. The selection, propagation, and promotion of superior varieties have emerged as key strategies for enhancing silkworm cocoon yield, quality, and economic returns. This requires mulberry to possess quantifiable traits such as strong leaf retention capacity, large leaf size, high biomass, and tolerance to pests, diseases, and environmental stresses such as drought, salinity, and cold. Therefore, the development of high-yielding, high-quality, and stress-tolerant superior strains adapted to local conditions has always been the fundamental goal of mulberry breeding [9,10].

Traditional mulberry breeding relies primarily on phenotypic selection, a time-consuming and inefficient process that limits the ability to meet rapidly changing demands. The rapid advancement of genomic technologies, particularly high-throughput sequencing, has enabled the analysis of mulberry genetic diversity at the molecular level, thereby accelerating breeding progress. In perennial woody plants, the traditional breeding process, from artificial hybridization to regional trials and official cultivar release, generally takes 10–15 years or longer [11]. In contrast, genomic selection enables early prediction of genomic estimated breeding values (GEBVs), allowing selection at the seedling stage and significantly shortening the breeding cycle while improving selection efficiency. Studies have shown that genomic selection can reduce the breeding cycle by approximately 30–50% while achieving prediction accuracies ranging from 0.6 to 0.8, significantly higher than those obtained through traditional phenotypic selection. As a perennial woody plant, mulberry also exhibits significant feasibility for genomic selection [12,13].

Genomics-driven breeding strategies can improve mulberry yield and quality while enhancing adaptability to environmental stresses, thereby providing strong technical support for sustainable agricultural development. Although mulberry genomics research is advancing rapidly, several challenges remain, including the efficient utilization of genomic data to guide practical breeding programs and the complexity of the mulberry genetic background. This review summarizes recent advances in mulberry genomics, examines the application potential of genomics in mulberry breeding, and discusses future research directions. These efforts aim to provide a scientific basis for the further development and upgrading of the mulberry industry.

## 2. Historical Development of Mulberry Genomics Research

Compared with major economic crops such as soybean, rice, and wheat, genomics research in mulberry began relatively late [14]. In 1990, Hironao Machii obtained transgenic mulberry plantlets by infecting mulberry leaf discs with *Agrobacterium tumefaciens* carrying the β-glucuronidase (*GUS*) reporter gene, demonstrating for the first time that mulberry leaf discs could accept foreign genes [15]. This finding was further confirmed in 1994 by Guan Zhiwen and colleagues through transformation experiments using a synthetic antimicrobial peptide D in mulberry leaf discs [16]. In the same year, Wang Yong and colleagues discussed the potential applications of genetic engineering in mulberry, highlighting its prospects for mulberry breeding [15]. In 1997, Lou Chengfu introduced transgenic technologies and their potential applications in mulberry, providing a foundation for subsequent genomic research [17]. With the advent of the 21st century, the rapid development of molecular sequencing technologies greatly advanced mulberry genomic research. Between 2011 and 2014, He Ningjia and colleagues reported that the implementation of the Mulberry Genome Project generated large-scale genomic resources, advancing cytological studies and providing a foundation for functional genomics research [18]. The first draft of the mulberry genome was released in 2013, marking a new stage in mulberry genomics research [19]. In 2014, Li Tian and colleagues developed MorusDB, an open-access mulberry genome database that provides extensive genomic and transcriptomic resources, the database integrates genome sequencing and assembly data, predicted genes and functional annotations, expressed sequence tags (ESTs), transposable elements (TEs), Gene Ontology (GO) terms, horizontal gene transfer information, and homologous and paralogous gene data, offering valuable resources for mulberry genomics research [20]. During this early stage of mulberry genomics, most studies relied on Illumina short-read sequencing combined with limited traditional physical mapping and mainly focused on the preliminary identification and functional characterization of gene families [21].

In 2022, Mukesh Jain and colleagues developed a high-quality genome sequence for Indian mulberry (*Morus indica*), achieving 96.5% completeness [22], by integrating multiple sequencing technologies, they revealed that approximately 49.2% of the genome consists of repetitive sequences and identified 27,435 high-confidence protein-coding genes [22]. This work represented a major leap forward, with third-generation long-read sequencing combined with Hi-C-assisted assembly becoming the standard approach. Long-read sequencing technologies, such as PacBio and Nanopore, helped resolve genome fragmentation issues caused by high heterozygosity. Meanwhile, resequencing of hundreds of mulberry accessions provided insights into their domestication histories and geographic origins. Research trends also shifted from single-gene identification toward genome-wide association studies (GWAS), enabling large-scale dissection of quantitative trait loci (QTLs) controlling yield and stress resistance [23].

Since 2023, mulberry genomics research has entered a phase of rapid expansion. Emerging technologies such as telomere-to-telomere (T2T) genome assembly, single-cell transcriptomics (scRNA-seq), and multi-omics data integration have enabled advances in pan-genome construction and haplotype-resolved analysis [24,25]. During this period, Liu Jiang and colleagues identified 16 members of the auxin response factor (*ARF*) gene family in mulberry. They conducted detailed analyses of their physicochemical properties and phylogenetic relationships, including amino acid length, molecular weight, hydrophilicity, and isoelectric point, providing a basis for functional and evolutionary studies of the *ARF* gene family as well as for genetic breeding [26]. Wei Jia and colleagues developed a haplotype-resolved genome assembly and identified disease-resistance genes in mulberry, highlighting the challenges posed by highly heterozygous genomes [27]. In the same year (2023), the mulberry research team at Southwest University reported the first gap-free mulberry reference genome, representing another milestone in mulberry genomics [28]. In 2024, Ma Bi and colleagues reviewed recent advances in mulberry genomics, including major progress in sequencing technologies, assembly strategies, and genome assembly quality, providing theoretical and technical references for future mulberry research [29].

Overall, the research paradigm in mulberry genomics is shifting from “genome decoding” toward “genome-enabled design breeding.”

## 3. Genomics-Based Approaches in Mulberry Breeding

### 3.1. Advances in Mulberry Stress-Resistance Breeding

Mulberry exhibits strong photosynthetic capacity, rapid growth, and notable tolerance to abiotic stresses such as drought, flooding, salinity-alkalinity, and cold. These adaptive traits are closely associated with its complex genetic background [30,31,32]. Studies have shown that mulberry varieties undergo natural sexual hybridization and cross-pollination and are characterized by high heterozygosity, genetic instability, abundant clonal variation, and multiple ploidy levels [33,34]. Japanese researchers have reported more than 120 naturally occurring triploid mulberry varieties, while Chinese scholars have identified over 60 natural triploids among cytologically characterized mulberry germplasm resources. In addition, mulberry exhibits mixoploidy, a rare phenomenon seldom observed in other plant species [35]. Different mulberry species exhibit substantial variation in chromosome number and ploidy level, ranging from diploid to 22-ploid. Such polyploidization significantly influences mulberry morphological traits, leaf structure, and nutritional quality [34]. Genome sequencing studies further indicate that transposable elements constitute a large proportion of the mulberry genome and play important roles in genome expansion, structural variation, and gene functional evolution. These elements are considered key factors underlying the complex genetic background of mulberry and contribute to its strong stress tolerance [36]. Based on existing genomic [19], metabolomic [33], and transcriptomic data [37], most of the key genes involved in the biosynthesis of various bioactive compounds in mulberry (*Morus notabilis*) have been identified. Furthermore, the regulatory pathways governing the synthesis of secondary metabolites in mulberry leaves and fruits have also been largely elucidated [36,37,38,39].

Epigenetic regulation also plays an important role in mulberry stress responses. Studies have shown that suppression of DNA methylation or silencing methyltransferase genes can enhance mulberry resistance to pathogenic infections [40]. In addition, analyses of mulberry genetic characteristics indicate that long-term evolutionary processes have led to the biosynthesis and accumulation of diverse phenolic and flavonoid metabolites. The regulatory networks controlling the biosynthesis of these compounds enhance mulberry defense responses against environmental stresses [41]. Currently, researchers have identified multiple genomic loci associated with important traits such as leaf size, leaf thickness, branching architecture, bud break timing, and disease resistance using GWAS and bulked segregant analysis (BSA). Notably, a tandem cluster of receptor-like protein kinase (*RLK*) genes associated with resistance to mulberry wilt disease has been identified. These genes show distinct expression patterns between resistant and susceptible varieties, providing potential targets for molecular breeding of disease resistance [42]. In 2019, Shuting Shang and colleagues cloned the mulberry osmotin gene (*MaOsm*) and analyzed its expression patterns [43]. The gene was expressed in roots, flowers, leaves, and buds, with particularly high expression in roots and flowers. Further analyses showed that *MaOsm* expression was significantly upregulated under abiotic stresses such as drought, salinity, abscisic acid (ABA) treatment, and cold, suggesting that this gene may play an important regulatory role in the environmental stress response [43]. In 2021, Sha Zheng and colleagues cloned the tryptophan decarboxylase gene (*MnTDC*) from *Morus notabilis* and overexpressed it in tobacco, which significantly enhanced the salt tolerance of transgenic plants [30]. Furthermore, accumulating evidence has demonstrated that multiple genes in mulberry play important roles in plant growth and development, signal transduction, abiotic stress responses, and the regulation of metabolic processes. These genes include Shaggy-like (SK) protein kinases [44], receptor for activated C kinase 1 (*RACK1*) [45], osmotin and osmotin-like proteins [46], encoding EIN3-like protein gene (*MnEIL3*) [47], responsible for regulating cellular pH and maintaining homeostasis gene (*MaNHX*) [48], dehydration-responsive protein gene (*MRD22*) [49], methionine sulfoxide reductase gene (*MMSR*) [50], 2-dehydro-3-deoxyphosphooctonate aldolase gene (*KdsA*) [51], and polyphenol oxidases gene (*Mn-PPO1*) [52] (Table 1).

Mulberry genetic research has gradually progressed from an early phase of genomic resource accumulation to a phase focused on the functional characterization of genes associated with key agronomic traits. This transition has revealed a coordinated adaptive cascade that involves environmental stress signals, epigenetic modifications (DNA methylation and small RNA regulation), transcriptome reprogramming (differential gene expression), and physiological and metabolic adaptation (antioxidant responses, photosynthetic optimization, and secondary metabolism). Concurrently, research on mulberry stress resistance has evolved from single-gene functional validation toward the integration of multi-omics approaches. Through the combined effects of epigenetic regulation, polyploid genomic advantages, and secondary metabolic regulatory networks, mulberry exhibits a highly coordinated defense system. Collectively, these findings provide a theoretical foundation for the future development of mulberry cultivars with enhanced stress resistance and improved quality.

### 3.2. Application of Gene-Editing Technologies in Mulberry Breeding

**Figure 1:**
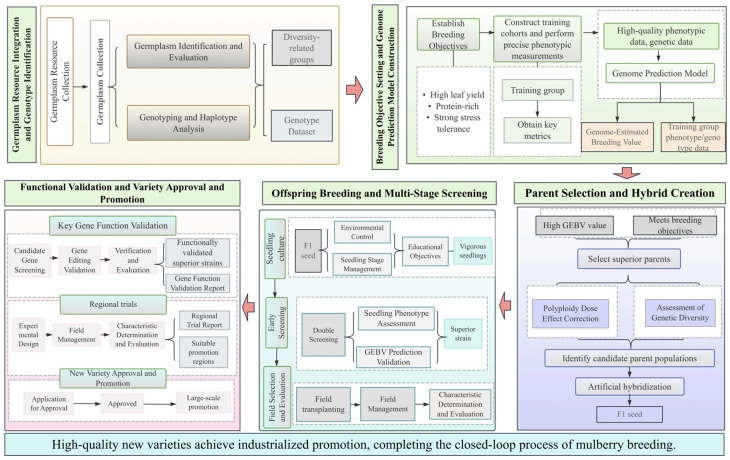
Detailed Workflow of Mulberry Genetic Breeding.

Here, mulberry breeding refers to the genetic improvement of mulberry through conventional breeding and genetic technologies. By employing approaches such as selection, hybridization, and modern biotechnological methods, new varieties with desirable traits, such as high leaf yield, superior fruit quality, enhanced stress tolerance, and disease resistance, can be developed [53,54]. Over time, a relatively well-defined mulberry breeding workflow has been established (Figure 1).

By the end of the 20th century, the development of improved mulberry varieties mainly relied on traditional breeding methods. These approaches primarily included hybrid breeding [55], polyploid breeding [56,57], and mutation breeding [58], which utilized natural variation and artificial selection to improve mulberry cultivars. Through these methods, a series of mulberry varieties with high yield, superior quality, and enhanced disease resistance were developed [59,60]. However, as a perennial woody plant, mulberry has biological characteristics such as a long reproductive cycle, high genetic heterozygosity, and extensive polyploidy, which lead to long breeding cycles, low efficiency, and difficulty in stably obtaining desired traits through conventional breeding [61]. Early studies indicated that although hybridization and selection could improve individual traits, breeding for complex quantitative traits, such as disease resistance, environmental adaptability, and nutritional quality, was often slow and unstable. This limitation highlighted the need for more precise breeding approaches, including molecular breeding and gene-editing technologies [62].

The introduction of molecular breeding and genome-assisted selection strategies has become an important complement to traditional breeding. For example, GWAS of multiple phenotypic traits in mulberry have identified molecular markers associated with agronomic characteristics such as leaf area and leaf weight, providing practical targets and a foundation for molecular breeding [63]. Nevertheless, even when molecular markers are applied, breeding still relies on naturally occurring or induced mutations and cannot directly create or precisely regulate specific genes or gene networks. To address this limitation, more precise genetic tools, particularly gene-editing technologies, have been developed [64].

In 2021, Huang Jin and colleagues reviewed the mechanisms of three major gene-editing technologies and their applications in plant breeding, analyzing their potential roles in mulberry functional genomics and genetic improvement and highlighting their capacity to promote more precise and efficient mulberry breeding [65]. Gene-editing technologies enable precise and targeted modification of specific genomic loci. Based on the type of site-specific nuclease used, gene-editing systems are generally classified into three categories: zinc finger nucleases (ZFNs) [66], transcription activator-like effector nucleases (TALENs) [67], and clustered regularly interspaced short palindromic repeats associated with Cas proteins (CRISPR/Cas) [68]. In addition, emerging approaches such as LEAPER RNA editing and retrotransposon-based editing have also been reported.

Among these technologies, CRISPR/Cas9 has created new opportunities for mulberry breeding. This system enables targeted cleavage and modification at specific genomic loci, allowing knockout, knock-in, or regulatory modification of target genes. Its efficiency and accuracy greatly exceed those of traditional breeding methods, enabling precise editing of mulberry genes and substantially improving the efficiency of genetic improvement [69]. For example, researchers used CRISPR/Cas9 to induce targeted mutations in the ferulate-5-hydroxylase (*F5H*) gene in paper mulberry. Multiple edited mutants were generated, and among 19 transgenic plants, 15 carried the target mutation, with editing efficiencies ranging from 22% to 68%. The mutation resulted in a 3.3-fold increase in G-type lignin content, significantly enhancing the adsorption properties of paper mulberry biomass-derived materials and thereby improving its biomass utilization value. More recently, CRISPR/Cas9 technology has been widely applied using sgRNA-guided Cas9 nucleases that generate double-strand breaks at specific DNA loci, enabling precise gene knockout or targeted mutation. This approach offers advantages such as high editing efficiency, operational simplicity, and the capacity for multiplex gene editing [70]. In addition, genome-wide identification and expression analyses of the *ARF* and *GATA* gene families in mulberry conducted by Liu Jiang and colleagues further highlight the importance of gene-editing technologies for elucidating the regulatory mechanisms underlying plant growth, development, and signal transduction [26,71].

Mulberry breeding is currently transitioning from traditional experience-based approaches to precise molecular breeding and gene-editing technologies. Although each strategy has distinct advantages and limitations, they are increasingly applied in complementary ways (Table 2). While gene-editing technologies have created new opportunities for mulberry breeding, several technical bottlenecks remain. On the one hand, the relatively weak in vitro regeneration capacity and low genetic transformation efficiency of mulberry make it difficult to establish stable and highly efficient transformation systems, which limits the broader application of genetic engineering and genome-editing technologies in mulberry breeding [72]. On the other hand, heterogeneity in cell division and mutation events during regeneration may produce chimeric plants containing cell populations with different genetic backgrounds, making it difficult to obtain stable and genetically uniform homozygous lines [73].

Traditional breeding has laid the foundation for mulberry improvement, whereas the integration of modern technologies such as gene editing is expected to enable the targeted improvement of important traits with greater precision, shorter breeding cycles, and lower costs.

## 4. Challenges and Future Directions in Mulberry Genomics and Breeding

### 4.1. Challenges in Mulberry Genomics Research

Despite significant advances in mulberry genomics in recent years, including whole-genome sequencing and the identification of genes associated with important agronomic traits, comprehensive genomic dissection remains challenging due to the intrinsic genomic complexity as a heteropolyploid species.

1. First, the high level of genetic heterozygosity and complex floral biology of mulberry make genomic sequencing and assembly technically demanding. In addition, sequencing of non-model plant genomes remains relatively costly, and obtaining high-quality genomic data often requires the integration of multiple sequencing technologies. Consequently, the development of high-yielding and highly resistant varieties through traditional hybrid breeding remains time-consuming and technically challenging.
2. Second, although progress has been made in mulberry genome sequencing, the regulatory networks controlling the expression of many functional genes remain poorly understood. In particular, genes associated with stress resistance and metabolic biosynthesis require further investigation. Current metabolomics research on mulberry involves the detection and analysis of a vast number of metabolites, presenting a high degree of complexity. The wide variety of naturally active metabolites in mulberry, coupled with their significant susceptibility to environmental factors, further complicates metabolomics research [74,75].
3. Furthermore, the chromosome number of mulberry has long been a subject of debate. Traditional research generally holds that the chromosome number is 2*n* = 28, whereas recent studies suggest it may be 2*n* = 14. This new understanding presents fresh challenges for research on the genetic evolution of mulberry and for molecular breeding [76].
4. Finally, public sharing of mulberry genomic data remains limited. Uneven distribution of research technologies and resources across countries and regions has also constrained the depth and breadth of mulberry studies. Collectively, these issues present challenges for mulberry genomics research while also highlighting new opportunities for further exploration of mulberry genetic resources.

### 4.2. Future Research Directions

#### 4.2.1. Integrative Multi-Omics Analyses

Since the release of the draft *Morus notabilis* genome in 2013, mulberry molecular biology research has entered a period of rapid development on a global scale. With continued progress, it has become increasingly clear that single-genome approaches are inherently limited, representing only the “tip of the iceberg” of species genetic diversity. Such approaches cannot fully capture structural variation, rare alleles, or adaptive differentiation at the population level and may overlook genetic information associated with important agronomic traits. These limitations restrict deeper investigations into mulberry breeding. Consequently, improving breeding efficiency and developing superior mulberry cultivars with desirable target traits to meet industrial demands have become major research priorities.

**Figure 2:**
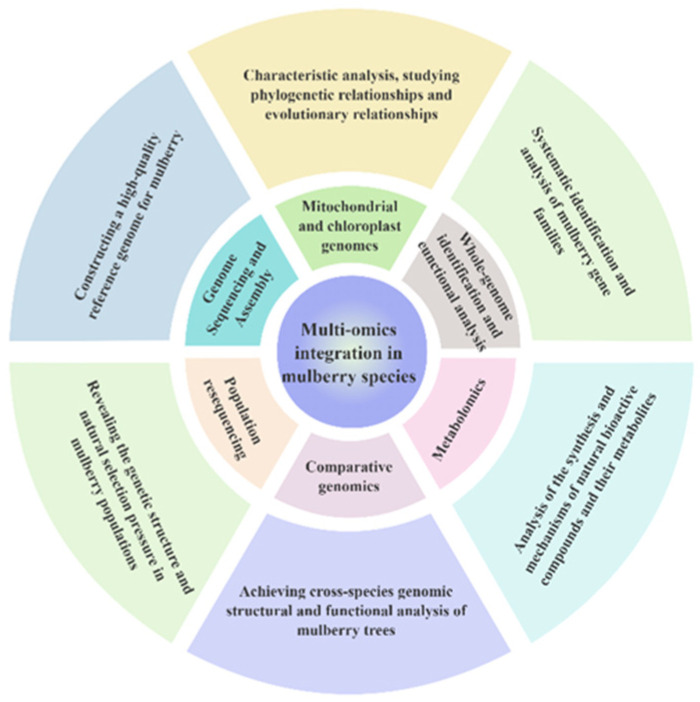
Multi-omics integration benefits diagram chart.

Recent advances in high-efficiency genome sequencing, genome assembly, and molecular marker technologies have enabled the construction of high-quality mulberry reference genomes, facilitating analyses of genetic diversity and phylogenetic relationships among mulberry germplasm resources. Additionally, analyses of mitochondrial and chloroplast genomes can help clarify species phylogeny and evolutionary relationships. Genome-wide identification approaches enable systematic characterization and functional analysis of mulberry gene families, while comparative genomics supports cross-species analysis of genome structure and gene function. Furthermore, population resequencing and pan-genome analyses provide comprehensive insights into the genetic structure of mulberry populations and the effects of natural selection. The integration of these approaches enables a more comprehensive understanding of mulberry genetic improvement and molecular breeding, thereby providing stronger technical support for the effective utilization and value enhancement of mulberry genetic resources (Figure 2).

However, the integration of multi-omics approaches in mulberry research remains at an early stage, and several challenges still need to be addressed.

- Difficulties in genome assembly and polyploid resolution: Conventional genome assembly methods often struggle to distinguish subtle differences among homologous chromosomes, making haplotype resolution particularly challenging. This limitation restricts the analysis of allele-specific expression and the molecular mechanisms underlying heterosis. Advanced technologies such as T2T genome assembly and haplotype-resolved sequencing are therefore required to improve assembly accuracy and genomic resolution.
- Bottlenecks in cross-omics data integration: Current studies often rely on the simple overlay of transcriptomic and metabolomic datasets, lacking deeper integration within a systems biology framework. For example, mulberry contains abundant secondary metabolites such as flavonoids and alkaloids, yet the dynamic feedback relationships between these metabolites and upstream gene expression (e.g., *PPO* genes) remain poorly characterized and have not yet been quantitatively modeled.
- Low genetic transformation efficiency and strong genotype dependency: Core candidate genes identified through omics analyses (such as *NHX* and *PPO*) are validated slowly in mulberry. Gene-editing and transgenic technologies exhibit strong genotype dependency, and many elite cultivars with available omics data cannot be stably transformed using current regeneration systems. The development of genotype-independent regeneration strategies, such as co-transformation with regeneration-promoting factors (e.g., BBM/Wus2), is therefore urgently needed.
- Complexity of environmental stress responses: Under natural conditions, mulberry plants are exposed to multiple interacting stresses. However, most omics studies are conducted under single-factor laboratory conditions, which cannot fully replicate the complex stress environments encountered in the field.

#### 4.2.2. Diversified Utilization of Mulberry Resources

The identification of stress-resistance genes and regulatory pathways in mulberry, followed by the breeding of stress-tolerant cultivars, is essential for improving tolerance to abiotic stresses such as low temperature, drought, flooding, and radiation. The ultimate goal is to expand the ecological adaptability and economic value of mulberry while promoting the sustainable development of the mulberry industry.

**Figure 3:**
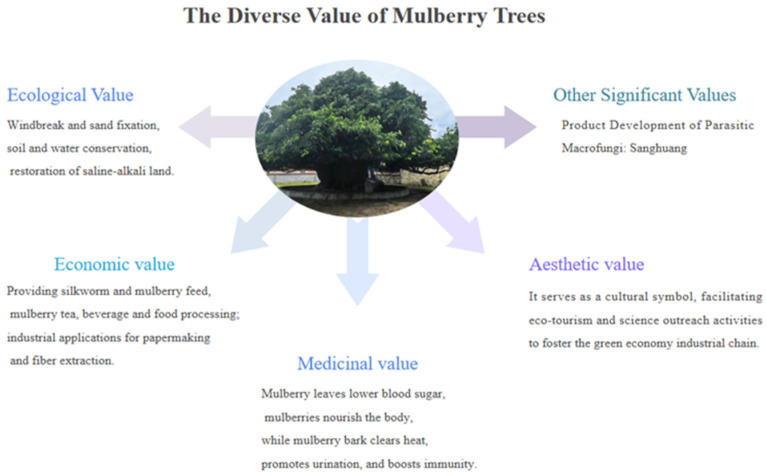
Diagram of mulberry economic uses chart.

**Figure 4:**
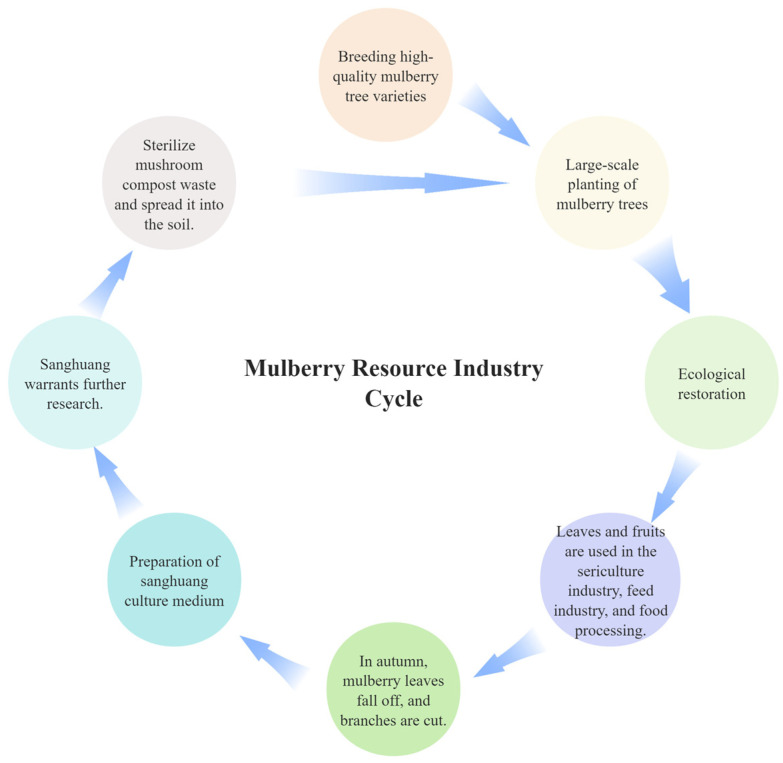
Schematic Diagram of Circular Development in the Mulberry Industry Economy.

1. Improving yield and quality while promoting product development: Mulberry has been cultivated for several thousand years. In recent times, substantial progress has been achieved in developing high-yield and high-quality mulberry varieties through modern breeding strategies, including polyploid breeding and molecular biotechnology such as genetic engineering. In terms of comprehensive resource utilization, mulberry leaves, characterized by high protein content and a balanced nutritional composition, have increasingly been developed as high-quality feed resources, supporting the coordinated development of the sericulture and livestock industries. In addition, mulberry leaves contain abundant bioactive compounds such as polyphenols and flavonoids with recognized health-promoting properties. These compounds enable further processing into products such as mulberry leaf tea and functional baked goods, expanding their applications in the functional food sector [77,78]. Mulberry fruits represent another important product of the mulberry and are rich in diverse nutrients and bioactive compounds. They can be processed into products such as wine, juice, and fructose, thereby increasing the added value of the mulberry industry in food processing [79]. Mulberry twigs, which are produced in large quantities during pruning and renewal, can also serve as substrates for cultivating edible and medicinal mushrooms, including shiitake, oyster mushroom, and *Fomes yucatanensis*. This application improves the utilization efficiency and economic value of mulberry twigs resources [80]. Additionally, mulberry twigs contain dihydromorin, morin, and sterol compounds, among which the flavonoid components exhibit significant antioxidant activity [81,82,83]. Clinical studies have shown that mulberry twigs have a favorable therapeutic effect on diabetes and its complications (such as arthropathy), effectively lowering blood glucose and alleviating related symptoms [84], thereby significantly enhancing the economic utilization value of mulberry twig resources. The root bark of *Morus alba*, known as Cortex Mori (*Sangbaipi*) in traditional medicine, exhibits a range of pharmacological activities, including hypoglycemic, anti-inflammatory, antioxidant, antitumor, cardioprotective, neuroprotective, and immunomodulatory effects. These properties provide opportunities for the development of functional pharmaceuticals and health-related products. Systematic exploration and utilization of the biological potential of different mulberry organs will therefore play an important role in promoting the development of related medicinal products and supporting public health (Figure 3).

2. Ecological restoration in environmentally fragile regions: Mulberries are native to the Yarlung Tsangpo River basin of the Qinghai–Tibet Plateau in China and exhibit strong tolerance to cold, drought, flooding, salinity–alkalinity, poor soils, and a wide range of environmental conditions [1,32]. The Qinghai–Tibet Plateau, with an average elevation exceeding 4000 m, is characterized by extreme climatic conditions and represents an important center of alpine plant diversification. Our research group has identified wild mulberry populations in the Naqu region of Tibet at elevations of approximately 3700 m, indicating a unique adaptation to plateau environments. The selection and improvement of superior mulberry varieties through genetic engineering for use in high-altitude and ecologically fragile regions therefore holds considerable potential for ecological restoration and sustainable land management.
3. Promoting a circular economy in the mulberry industry: Historically, the mulberry–fish pond system represented a classical model of circular agriculture. Building on this concept, a modern mulberry-based circular development model can be established. Taking the cultivation of the medicinal fungus *Fomes yucatanensis* Sanghuang as an example, we propose a “mulberry–silkworm–fungus–fertilizer–mulberry” industrial cycle (Figure 4).

Within this framework, molecular biology and molecular breeding technologies are employed to develop mulberry cultivars with high yield and strong stress tolerance, enabling large-scale cultivation in ecological restoration areas. First, marker-assisted selection (MAS) is applied to identify mulberry varieties with high biomass and broad environmental adaptability. Second, targeted regulation of mulberry leaf metabolite profiles (e.g., alkaloids and flavonoids) and loci associated with protein synthesis enhances the value of mulberry as a feed and functional food resource. In addition, during the preparation of fungal cultivation substrates from mulberry twigs, metabolomic evaluation and genomic analyses focus on lignocellulose composition and key biosynthetic genes such as *CAD* and *COMT*. These approaches aim to optimize lignocellulose structure, improve the bioconversion efficiency of medicinal fungi, and accelerate the degradation of spent substrates after soil incorporation. Finally, improvement of genes associated with root system architecture (RSA) and nitrogen use efficiency (NUE) enhances nutrient uptake from recycled fungal fertilizers, reducing chemical fertilizer inputs while simultaneously improving soil properties and ecological stability.

This model represents not merely an industrial integration but rather a coordinated system that combines mulberry molecular breeding, resource utilization, and ecological recycling. It provides a sustainable framework for efficient resource use, high-value product development, and ecological restoration, offering considerable potential for large-scale application in the modern mulberry industry.

## 5. Conclusions

Recent advances in mulberry genomics have significantly improved breeding efficiency and precision while providing important technological support for the transition toward a circular bioeconomy and the restoration of fragile ecosystems. Comprehensive analysis of the mulberry genome enables the identification of genes associated with growth, stress resistance, and yield, facilitating the development of high-yielding, high-quality cultivars adapted to diverse environmental conditions.

However, mulberry breeding research remains relatively fragmented, and several key scientific questions remain unresolved. Compared with major crops such as rice and soybean, the functional characterization of mulberry genes is still limited. Future research should focus on constructing high-quality mulberry genomes, identifying structural variations (SVs) among populations, and elucidating allele dosage effects in polyploid mulberry species to support more precise genome editing. In addition, integrating modern breeding technologies with genomic selection (GS) could further shorten breeding cycles and improve selection efficiency. The application of systems biology approaches to investigate the “mulberry–silkworm–fungus–fertilizer–mulberry” interaction model, together with multi-omics strategies such as polyploid genome assembly, comparative genomics, and pan-genomics to strengthen research on trait-gene associations, will accelerate mulberry research progress and contribute to the sustainable development of the mulberry industry.

## Figures and Tables

**Table 1:** Molecular detection and phenotypic effects of mulberry stress-resistance genes.

Gene	Host	Inducing Factors	Molecular Evidence	Phenotypic Effects
*MnSK*	Morus alba	Drought	The expression of *MmSK* in mulberry was up-regulated under various abiotic stress treatments. Meanwhile, we observed higher expression levels in the phloem, contrasted with other tissues.	As drought stress duration increased, the soluble protein content, proline content and malondialdehyde (MDA) content gradually increased. These results suggested that the *MmSK* gene could function as a positive regulator of drought stress in mulberry.
*RACK1*	mulberry G-protein genes	Drought and salt stresses	qRT-PCR confirmed the expression of *MaGα*, *MaGβ*, *MaGγ1*, and *MaGγ2* in the receptor system.	Under drought and salt stress, seed germination rates were significantly increased; leaf water loss rates were reduced and chlorophyll content remained higher, Further analysis showed that mulberry G-proteins may regulate drought and salt tolerances by modulating reactive oxygen species’ detoxification.
Osmotin and osmotin-like proteins	*Morus indica* cv. K2	Fungal infection, Salinity and drought	Osmotin was evaluated for its effect on abiotic stress tolerance with a constitutive as well as a stress-inducible promoter, and the transgenic plants showed a better performance than the non-transgenic plants.	The transgenic plants with the stress-inducible promoter were able to tolerate salt and drought stress more efficiently than those with the constitutive promoter.
*MnEIL3*	Mulberry, overexpression in *Arabidopsis*		Arabidopsis overexpressing *MnEIL3* exhibited an enhanced tolerance to salt and drought stresses, identified as an ethylene-responsive transcription factor.	*MnEIL3* overexpression in Arabidopsis significantly upregulated the transcript abundances of ethylene biosynthetic genes. Furthermore, *MnEIL3* enhanced the activities of the *MnACO1* and *MnACS1* promoters, which respond to salt and drought stresses. Thus, *MnEIL3* may play important roles in tolerance to abiotic stresses and the expression of ethylene biosynthetic genes.
*MaNHX*	Mulberry	Salt, drought abscisic acid and other signal molecules	Transcriptome data and real-time PCR identified seven members of the NHX gene family. These genes exhibit complex expression patterns under salt, drought, and signaling molecule treatments	*MaNHXs* not only could be induced by salt, drought and abscisic acid, as described in the literature, but were also induced by other signal molecules, which indicated that *MaNHX* members exhibited diverse and complicated expression patterns in different mulberry tissues under various abiotic stresses
*MRD22*	Mulberry	Drought, low temperature and salt stresses	Northern blot analysis showed that expression peaked within 2 h of water loss.	Encodes a dehydration-responsive protein that delays plant wilting under cold stress.
*MMSR*	Mulberry	Drought and salt stresses	A full-length cDNA sequence encoding methionine sulfoxide reductase (MSR) from mulberry, qRT-PCR analyses indicated enhanced expression in old leaves and damaged tissues.	*MMSR* is a key component in mulberry response to abiotic stress, potentially enhancing their stress resistance by scavenging reactive oxygen species (ROS) and repairing oxidatively damaged proteins, thus prolonging leaf lifespan.
*KdsA*	Mulberry	High salinity; drought	qRT-PCR analysis showed downregulation of gene expression under stress conditions.	Involved in cell wall pectin biosynthesis; reduced expression leads to thinner cell walls under drought stress and decreased biomass accumulation.
*Mn-PPO* *s*	*Morus notabilis*	UV radiation, drought, and Botrytis cinerea infection	qRT-PCR revealed expression patterns: significantly upregulated under UV treatment; most PPO genes first increased and then decreased under drought; MnPPO1 was strongly induced after pathogen infection.	The products derived from the oxidation of chlorogenic acid by *MnPPOs* were tested for antimicrobial activity. five *MnPPOs* exhibited good inhibitory effects against *Escherichia coli*, *Pseudomonas aeruginosa*, *Staphylococcus aureus*, *Sclerotinia sclerotiorum*, and *Botrytis cinerea*. The products of *MnPPO1* increased cell membrane permeability and chitinase and β-1,3-glucanase activities.

**Table 2:** Comparison between traditional breeding approaches and modern genomic technologies.

Comparative Dimensions	Traditional Breeding Approaches	Modern Genomic Technologies
Underlying principle	Exploits natural genetic variation through artificial selection, relying on genetic recombination and spontaneous mutation	Utilizes molecular and genomic tools to achieve precise, targeted modification of genetic information
Breeding efficiency	Characterized by long breeding cycles and relatively low efficiency, dependent on extensive phenotypic screening	High efficiency and accuracy, enabling rapid identification and improvement of target traits
Trait targeting	Limited precision in trait selection, with outcomes largely influenced by genetic randomness	High precision in trait selection, allowing targeted genetic improvement
Technical complexity	Methodologically straightforward and easy to implement	Technically complex, requiring advanced instrumentation and specialized expertise
Application Scope	Appropriate for long-term genetic improvement and conventional breeding programs	Particularly suitable for rapid breeding and the improvement of complex or quantitative traits
Public acceptance	Generally well accepted, with few regulatory or ethical concerns	Acceptance varies among regions and is often influenced by regulatory, ethical, and biosafety considerations

## Data Availability

Data are contained within the article.
